# Mesenchymal stem cell-derived exosomal miR-223 regulates neuronal cell apoptosis

**DOI:** 10.1038/s41419-020-2490-4

**Published:** 2020-04-27

**Authors:** Hong Wei, Yuhao Xu, Qi Chen, Hui Chen, Xiaolan Zhu, Yuefeng Li

**Affiliations:** 1 Department of Central Laboratory, The Fourth Affiliated Hospital of Jiangsu University, Zhenjiang, Jiangsu 212001 China; 2 Jiangsu University, Zhenjiang, Jiangsu 212003 China; 3 Department of Neurology, The Affiliated Hospital of Jiangsu University, Zhenjiang, Jiangsu 212001 China; 4 Department of Radiology, The Affiliated Hospital of Jiangsu University, Zhenjiang, Jiangsu 212001 China

**Keywords:** Cell death in the nervous system, Mesenchymal stem cells

## Abstract

Hypoxia limits the survival and function of neurons in the development of Alzheimer’s diseases. Exosome-dependent intercellular communication is an emerging signaling mechanism involved in tissue repair and regeneration; however, the effect and underlying mechanism of mesenchymal stem cell-derived exosomes in regulating neuronal cell apoptosis have not been determined. Here, we showed that the establishment of an AD cell model was accompanied by increased HIF-1α expression and cell apoptosis, impaired cell migration, and decreased miR-223. MSC-derived exosomes were internalized by the AD cell coculture model in a time-dependent manner, resulting in reduced cell apoptosis, enhanced cell migration and increased miR-223, and these effects were reversed by KC7F2, a hypoxic inhibitor. Furthermore, MSC-derived exosomal miR-223 inhibited the apoptosis of neurons in vitro by targeting PTEN, thus activating the PI3K/Akt pathway. In addition, exosomes isolated from the serum of AD patients promoted cell apoptosis. In short, our study showed that MSC-derived exosomal miR-223 protected neuronal cells from apoptosis through the PTEN-PI3K/Akt pathway and provided a potential therapeutic approach for AD.

## Introduction

Alzheimer’s diseases (AD) is an irreversible neurodegenerative disease with complex pathogenesis, affecting 47 million people worldwide^1^. The high mortality of patients with AD is mainly ascribed to the complex aetiology and limited therapeutic interventions. In the past few years, based on the abnormal aggregation of Aβ, researchers have attempted to find methods to inhibit the aggregation and spread of Aβ protein or to promote its clearance^2^. However, these drug candidates failed to reverse AD or delay progression. Apart from the presence of Aβ, cerebral hypoxia, which is a high-risk factor for AD, could contribute to the cellular and tissue damage that leads to cell dysfunction and cellular aging followed by cognitive and memory deficits^3^. In addition, various studies have revealed that hypoxia plays a role in accelerating the amyloidogenic process of APP, resulting in Aβ accumulation^4^. Nevertheless, the relationship between hypoxia and Aβ accumulation remains unexplored.

Mesenchymal stem cell (MSC) is a unique stromal cell type that confers functional and structural benefits in neurodegenerative diseases, including AD^5^. Accumulating evidences have proven the therapeutic effects of MSCs due to their unique properties, including comparatively easy access, rapid proliferative capacity, and multipotency. Recent data also indicated that MSCs could benefit AD via their paracrine effect^6^, although obstacles remain to be overcome, particularly poor cell survival, and histocompatibility^7^. Exosomes are thought to be a potent secretory product of MSCs containing a specific payload of small RNAs and protein, which play an important role in MSC-mediated biological effects^8^. Exosomes derived from MSCs are nano-sized vesicles 30–120 nm in diameter that originate from specific cells^9^ and can mediate neuronal protection and nerve regeneration, which may slow down or even reverse the progression of AD. Moreover, MSC-derived exosomes were shown to carry certain molecules that yield tissue-protective effects in stroke models following neural injury^10^. In a glioma model in vivo, Katakowski et al. reported a promising protective effect following direct intra-tumoral injection of MSC-derived exosomes^11^. Hence, MSCs can potentially better protect nerve cells through their exosomal cargo than the MSCs themselves.

MicroRNAs, which post-transcriptionally control the translation and stability of mRNAs, are an essential component of the exosomal cargo^5^. A study confirmed that miR-223 is selectively packed into exosomes^12^, and exosome-encapsulated miRNAs can be stably stored by avoiding nuclease degradation^13^. MiR-223 has emerged as a putative predictor of neuroprotection, and it was reported to be able to limit inflammation to prevent collateral damage during infection and to prevent oncogenic myeloid transformation^14^. In our study, miR-223 was found to be associated with PTEN, which controls multiple cellular responses, such as apoptosis, as a critical regulator of the PI3K/Akt signal transduction pathway^15^.

Our previous study confirmed that miR-223 was selectively packed into exosomes and was significantly different between AD patients and healthy controls, and it may interfere with the progression of AD as a protective factor^16^. In this study, we aimed to explore whether MSC-derived exosomes have protective effects on neuronal apoptosis and whether this effect is miR-223 content dependent.

## Materials and methods

### Establishment of the AD model

Cultured human neuroblastoma SH-SY5Y cells were purchased from the Chinese Academy of Sciences. When the SH-SY5Y cells grew to 80%, the experiments were started. SH-SY5Y cells were resuspended to the appropriate concentration, and amyloid β protein fragment 1–40(Aβ_1–40_) (#A1075, Sigma-Aldrich, St. Louis, MO, USA) was added to the fresh complete culture medium up to a final concentration of 5 μmol/L. Cells were incubated in the cell culture incubator with 5% CO_2_ at 37 °C for 24 or 48 h.

### Preparation, culture and identification of MSCs

MSCs were isolated from human umbilical cords following previously described methods after informed consent was obtained according to institutional guidelines under the approved protocol^17^. MSCs were cultured in DMEM/F-12 (#11320033, GIBCO BRL, Grand Island, NY, USA) containing 10% foetal bovine serum (#10099141C, GIBCO BRL) and 1% penicillin/streptomycin (#15240062, GIBCO BRL). MSCs between passages 5–8 were used for subsequent experiments. To confirm the identity of the cells, the phenotypic profile of MSCs was evaluated by flow cytometry analysis using PE-labeled human anti-CD29 (#102216, Biolegend, San Diego, CA, USA), anti-CD44 (#338804, Biolegend), anti-CD90 (#328109, Biolegend), anti-CD11b (#101210, Biolegend) or anti-CD45 antibody (#103106, Biolegend). The morphology was observed by inverted microscopy. MSCs were cultured in MSC osteogenic differentiation medium for differentiation, and differentiated cells were identified by Alizarin Red staining (#G8550, Solarbio, Beijing, China) and Oil Red O staining (#O8010, Solarbio).

### Participants and sample collection

The enrolled patients were described in our previous study^16^ and the related information is shown in additional Table 1. To reduce experimental error, we mixed exosomes derived from two patient sera as one sample and each group was made up of three samples. The experimental protocol was approved by the institutional review board with informed consent from the patients.

### Preparation and characterization of exosomes

Exosomes were isolated from MSCs or serum as previously described. The isolation method comprised an additional centrifugation step to remove small cell debris followed by ultracentrifugation at 100,000 × *g* for 1 h to generate an exosome pellet. Afterwards, the pelleted exosomes were resuspended in PBS. The concentration and size distribution of exosomes were confirmed by Nanoparticle Tracking Analysis (NTA) using NanoSight NS300. The morphology was observed by transmission electron microscopy (TEM). To detect exosome markers and negative markers, Western blotting was performed with anti-CD63 (#ab59479, Abcam, Cambridge, MA, USA), anti-CD81 (#ab79559, Abcam) and anti-tubulin (#ab6160, Abcam) antibodies.

### MSCs or exosomes treatment

In total, 1 × 10^5^ SH-SY5Y cells were seeded into the lower chambers. For the cell treatment, approximately 5 × 10^5^ MSCs were seeded into the upper chambers of 6-well cell culture inserts. Exosomes were added to the culture medium at 2 μg of exosomes per 1 × 10^5^ recipient cells. The N-SMase inhibitor GW4869(20 μM) (#D1692, Sigma-Aldrich) was added to MSCs.

### Cell transfection and hypoxia preconditioning

MSCs were plated on 6-well plates and transfected using Lipofectamine 2000 (#11668019, Invitrogen, Carlsbad, CA, USA) in accordance with the manufacturer’s instructions. After 48 h, the cells were collected for further study. The in vitro preconditioning hypoxia model was established by flooding the chamber with 95% N_2_ and CO_2_ as described previously. The HIF-1α inhibitor KC7F2 (#S7946, Selleck Chemicals, Houston, TX, USA) was used before some experiments.

### Western blot assay

Total protein was isolated with RIPA lysis buffer (#R0020, Solarbio). Approximately 10 μg of protein was separated in 12% gels by sodium dodecyl sulfate-polyacrylamide gel electrophoresis, and then transferred to a PVDF membrane. The membrane was blocked with 5% bovine serum albumin at room temperature for 2 h and then immunoblotted with antibodies against Aβ (#ab62658, Abcam), HIF-1α (#ab51608, Abcam), PTEN (#ab32199, Abcam) and p-AKT (#ab8805, Abcam). Chemiluminescence was detected with the ChemiDoc MP imager.

### RT-PCR

Total RNA was isolated using Trizol reagent. cDNA was synthesized using a FastQuant RT Kit (with gDNase) (#KR106, Tiangen, Shanghai, China) according to the manufacturer’s instructions. Quantitation of miRNAs was carried out using a miRcute Plus miRNA qPCR Detection Kit (#FP411, Tiangen). The raw RT-qPCR miRNAs data were normalized to the spiked U6 snRNA levels as described previously^18^. The quantitative PCR procedures were carried out with real-time PCR SYBR Green q-PCR Super-mix. The miRNA expression levels were analyzed and quantified by calculating using the 2^−ΔΔCt^ method.

### Confocal microscopy

The exosomes from MSCs were labeled with PKH67 (#PKH67GL, Sigma-Aldrich) according to the protocol. After treatment with the indicated conditions for 0, 24 or 48 h, SH-SY5Y cells were washed with PBS and fixed with 4% paraformaldehyde for 30 min. Then, the cells were permeabilized with 0.5% Triton X-100, and the reaction was stopped by 5% bovine serum albumin. The cells were fixed and stained with DAPI. The uptake of labeled exosomes by the AD model was detected by a Leica TCS SP5 II laser scanning confocal microscope.

### Flow cytometry

In each of the experiments, SH-SY5Y cells were seeded in 6-well plates and treated with the indicated conditions for 48 h. Cell apoptosis was detected using an Annexin V/PI detection kit (#559763, BD Biosciences, San Jose, CA, USA) with a FACS Calibur flow cytometer, and data were analyzed using FlowJo software.

### Measurements of IL-1β, IL-6, TNF-α, and CRP concentrations

The concentrations of IL-6, IL-1β, TNF-α, and CRP were detected with ELISA kits (#E01I0006, #E09I0010, #E03T0008, #E01C0009, Blue gene, Shanghai, China).

### Wound healing assay

The AD model cells were seeded at a density of 1.2 × 10^4^ cells/well in a 6-well plate, maintained at 37 °C and 5% CO_2_ overnight and starved the next day with no FBS medium for 24 h. The cell monolayer was scratched with a 200 μl pipette tip as described previously. Cells were washed with PBS and incubated with 5 × 10^8^ EVs/ml in 1% EV-depleted FBS medium for 48 h.

### Experimental design

The schematic illustration of the experiment was shown in the additional file 1.

### Statistical analysis

All experiments were repeated at least three times. Data were analyzed with GraphPad Prism 6.0. Student’s *t*-test was used to determine significant differences between two groups, and one-way ANOVA test with Tukey’s post hoc analysis was performed to compare more than two groups. All data are reported as mean ± SD and a value of *P* < 0.05 was considered statistically significant.

## Results

### The establishment of the AD cell model was accompanied by hypoxia

**Fig. 1:**
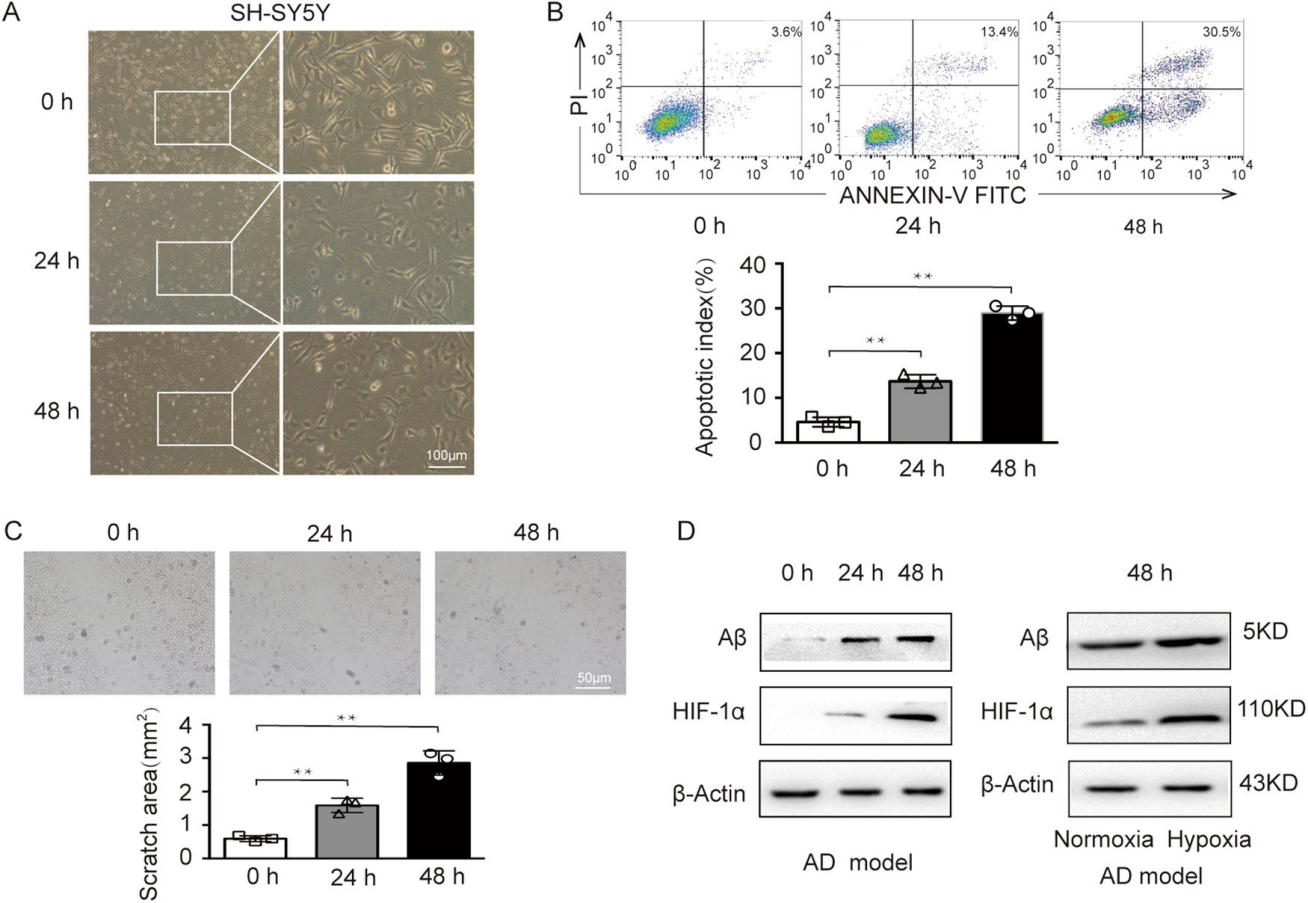
The establishment of the AD model was accompanied by hypoxia. **a** Morphological characteristics of SH-SY5Y cells treated with Aβ_1–40_ for 0, 24 or 48 h. **b** Apoptosis of the AD model was assessed. **c** Cell migration ability was analyzed by wound healing assay, and the scratched area was quantified with ImageJ in SH-SY5Y cells exposed to Aβ_1–40_ for 0 or 24 h. **d** Protein levels of HIF-1α and Aβ in the SH-SY5Y cells exposed to Aβ_1–40_ for 0, 24 or 48 h (left) and changes in HIF-1α and Aβ in the AD model exposed to normoxia or hypoxia (right); **p < 0.01.

The AD model was established with Aβ_1–40_ as previously reported^19,20^. The first approach to validate our model was to evaluate the morphology of the cells. Normally, SH-SY5Y cells are large and bright and round or oval in shape. After exposure to Aβ_1–40_, the cell number was reduced, the morphological appearance was changed correspondingly, and cell body shrinkage, rough cell surface, and retracted cell neurites were observed. As the exposure time increased, the morphological changes became more obvious (Fig. 1a). The flow cytometry results showed a significant, time-dependent increase in cell apoptosis in the AD model (Fig. 1b). In the wound healing assay, after Aβ_1–40_ exposure, the scratch area was significantly bigger (Fig. 1c), which may be due to the increased Aβ in the cell limiting the migration of neurons. The pretreated cells showed changes in their morphology and physiological function. Next, the existence of a hypoxic microenvironment in the AD model was determined. As expected, HIF-1α was elevated in the AD model in a time-dependent manner, which indicated increasing hypoxic stress. Subsequently, we determined whether there is a correlation between hypoxia and Aβ. We established an AD model under normoxic and hypoxic conditions and found that hypoxia enhanced the expression of Aβ (Fig. 1d). In conclusion, the AD model with SH-SY5Y cells was successfully established by Aβ_1–40_, and this AD model operated under hypoxic conditions.

### The effect of MSC-derived exosomes on the AD model

**Fig. 2:**
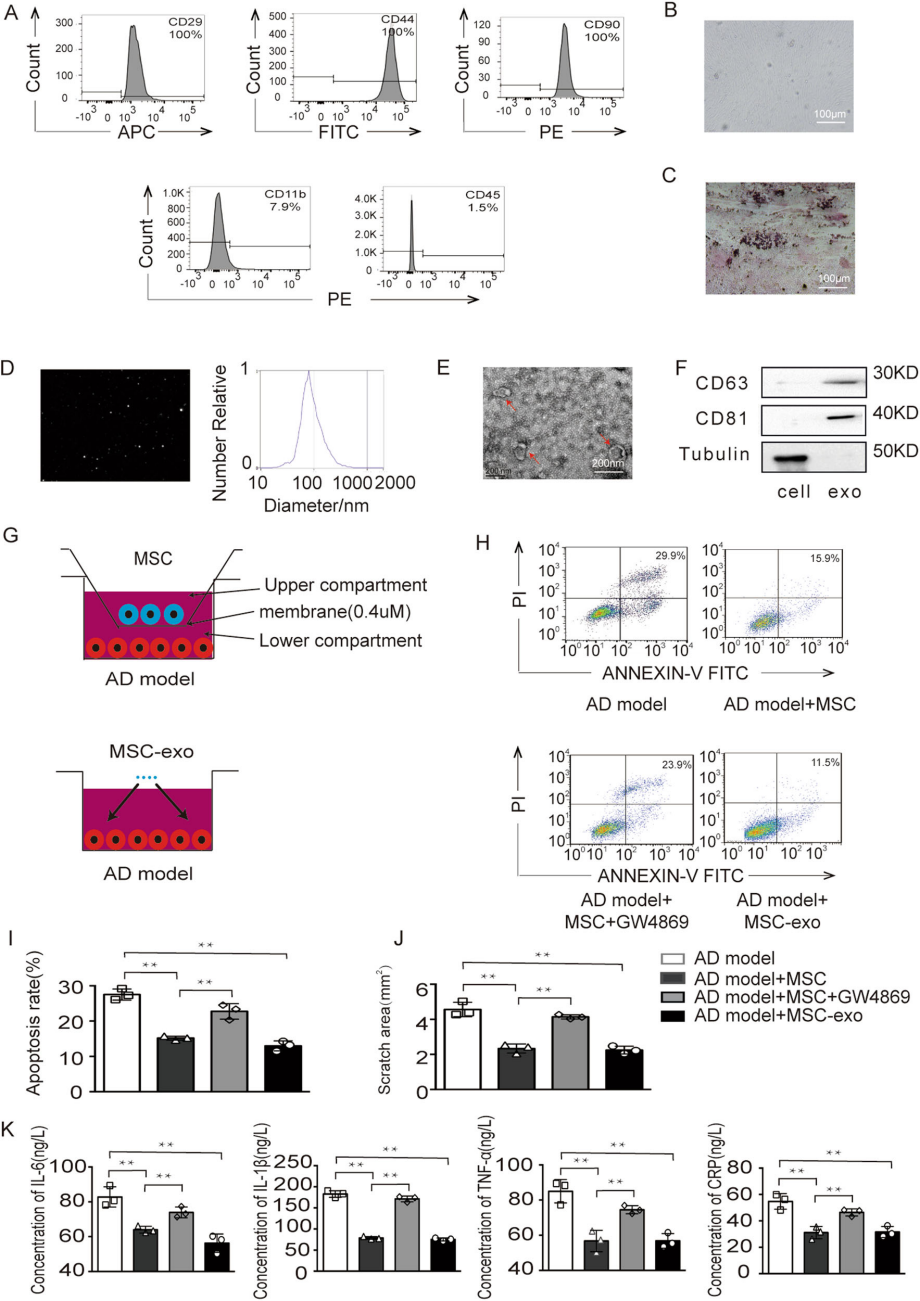
Exosomes derived from MSCs had a protective effect on the AD model. **a** Flow cytometry analysis of the surface markers of MSCs showed that the MSCs were positive for CD markers such as CD29, CD44 and CD90 and negative for CD11b and CD45. **b** The morphology of MSCs was observed under a microscope. **c** Representative images of osteocyte differentiation of MSCs cultured in the differentiation media. **d**–**f** Exosome characterization was performed by NTA, electron microscopy and Western blotting. **g** A Transwell non-contact coculture assay system was used with MSCs and the AD model, and a contact coculture assay system was used with exosomes and the AD model. **h**, **i** The apoptosis ratio of the AD model in the four groups was detected. **j** The scratched area was quantified with ImageJ of the four groups. **k** Levels of the inflammatory cytokines IL-6, IL-1β, TNF-α, and CRP in cell supernatants from the four groups were measured; **p < 0.01.

To analyze the impact of MSC-derived exosomes on the AD model, we first characterized MSCs and their derived exosomes. The MSCs were successfully isolated from human umbilical cords and were identified by molecular markers, morphological observation and differentiation ability. MSCs positively expressed the cell surface markers CD29, CD44 and CD90 but negatively expressed CD11b and CD45, as previously reported^21^ (Fig. 2a). The cell morphology of MSCs was observed under an inverted microscope with fibroblast-like long spindles in an orderly arrangement (Fig. 2b). Then, the multilineage differentiation capability of MSCs was analyzed based on their osteogenesis ability. Positive cytochemical staining was observed after osteogenic induction of MSCs (Fig. 2c). Subsequently, exosomes derived from MSCs were analyzed for size distribution and numbers with a NanoSight system. NTA indicated a homogenous population with low dispersity and with a peak in particle size at ~90 nm (Fig. 2d). In addition, a bilayer cup-shaped morphology was detected (Fig. 2e). Western blot analysis showed the expression of the exosomal marker proteins CD63 and CD81, while the expression of tubulin was negative (Fig. 2f). Therefore, the above results demonstrated the efficacy of the extraction protocol.

Once secreted, exosomes are internalized by neighboring or distant cells. To determine the effect of exosomes, we employed a Transwell coculture system for MSCs and an AD model in which the cells were separated by a porous membrane with 0.4 μM pores. Additionally, a contact coculture system for exosomes and an AD model was established (Fig. 2g). The AD model was treated with MSCs or exosomes derived from MSCs. As expected, the apoptosis rate of the AD model cocultured with MSCs and exosomes was significantly reduced (Fig. 2h, i). Similarly, we found that MSCs and their exosomes decreased the scratch area in the wound healing assay (Fig. 2j and additional file 2). In addition, we assessed the concentrations of IL-6, IL-1β, TNF-α, and CRP. As shown in Fig. 2k, the concentrations of inflammatory factors in MSCs and exosomes were significantly reduced. However, these protective effects were inhibited after pretreatment with the exosome inhibitor GW4869. These findings indicated that exosomes protected the AD model against the damaging effect. In summary, MSCs and exosomes derived from MSCs showed similar neuroprotective effects.

### Internalization of MSC-secreted exosomes by the AD model

**Fig. 3:**
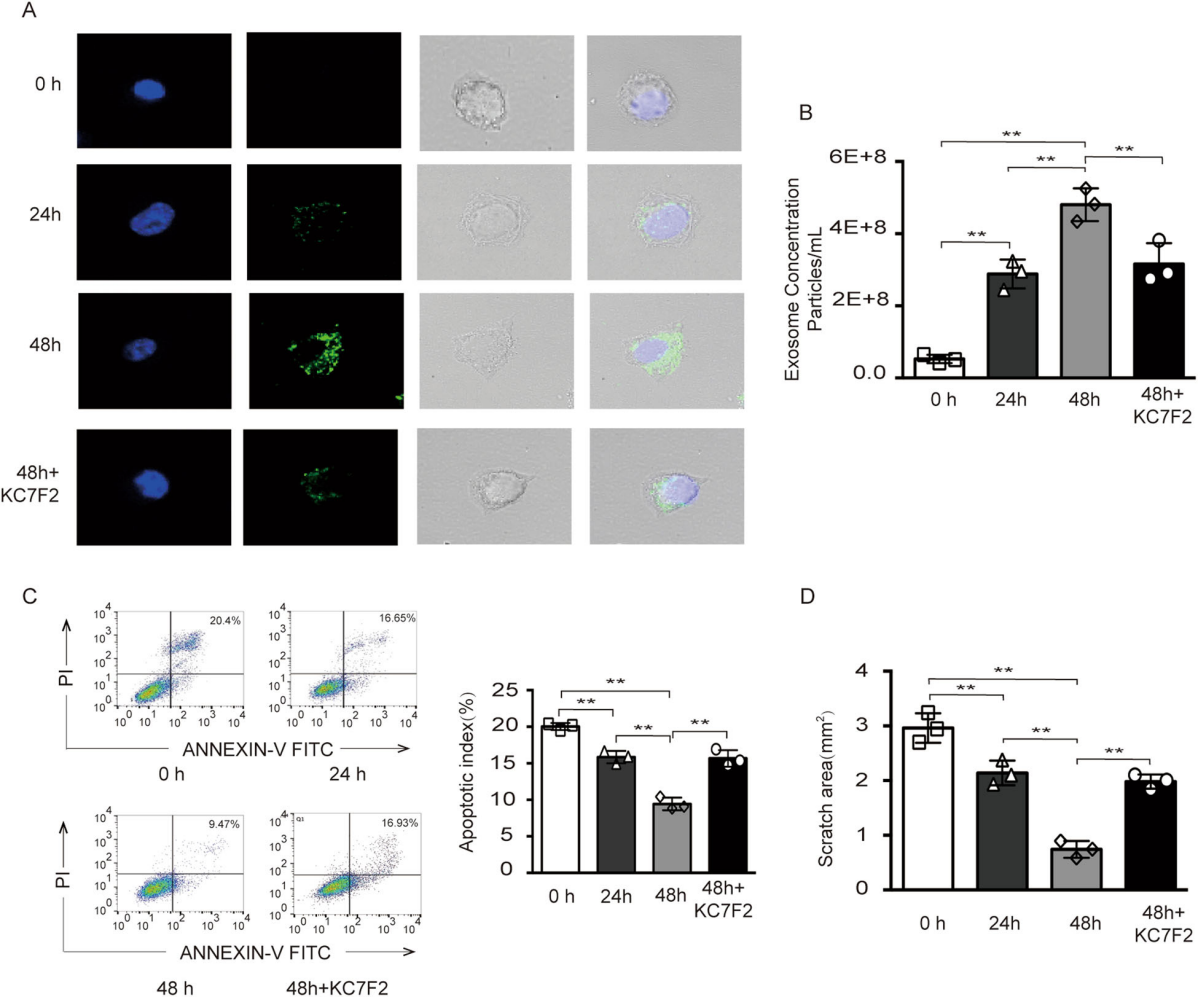
Internalization of MSC-secreted exosomes by the AD model. **a**, **b** The internalization of PKH-67-labeled exosomes and concentration of exosomes were analyzed in the AD model cocultured with exosomes for 0, 24, 48 h with or without KC7F2 treatment, respectively. **c**, **d** Cell apoptosis, the related quantitative analysis of apoptosis and the scratched area was analyzed in the AD model; **p < 0.01.

To verify the function of MSC-derived exosomes in the AD model, MSC-secreted exosomes were labeled with PKH-67. To determine the uptake efficiency in the AD model, we treated the AD model with equal amounts of labeled exosomes for 0, 24 or 48 h and observed significant uptake of exosomes by the AD model. With the extension of coculture time, exosomes were increased to a greater extent under 48 h of coculture. To explore whether the hypoxic environment in the AD model enhanced exosome internalization, the hypoxic inhibitor KC7F2 was added to the AD model, and the internalization of exosomes was reduced compared to that in the no KC7F2 group. The differences in the group at 24 h and treatment with KC7F2 for 48 h were not significant (Fig. 3a). As expected, exosome quantification was increased with of the increase in coculture time and reduced in the group treated with KC7F2 for 48 h (Fig. 3b). Next, we assessed whether the MSC-derived exosomes internalized by the AD model would affect their function. We found that apoptosis in the AD model treated with exosomes was significantly reduced, while apoptosis in the AD model for 48 h and pretreatment with KC7F2 was increased compared with that in the no KC7F2 group (Fig. 3c). In addition, a wound healing assay was performed to assess the migrate ability of nerve cells. As shown, the scratched area was significantly reduced in the AD model pretreated with exosomes derived from MSCs, while it was increased in the group treated with KC7F2 (Fig. 3d and additional file 3). As expected, as the coculture time was prolonged, the ability of cells to protect against apoptosis increased, and cell migration was enhanced, which may be due to the increase in exosomes internalized by the AD model. This effect was significantly reduced while hypoxic conditions were inhibited. In summary, internalization of MSC-derived exosomes by the AD model was enhanced via hypoxia.

### MSC-derived exosomal miR-223 reduces cell apoptosis in an AD model by targeting the PTEN-PI3K/Akt pathway

**Fig. 4:**
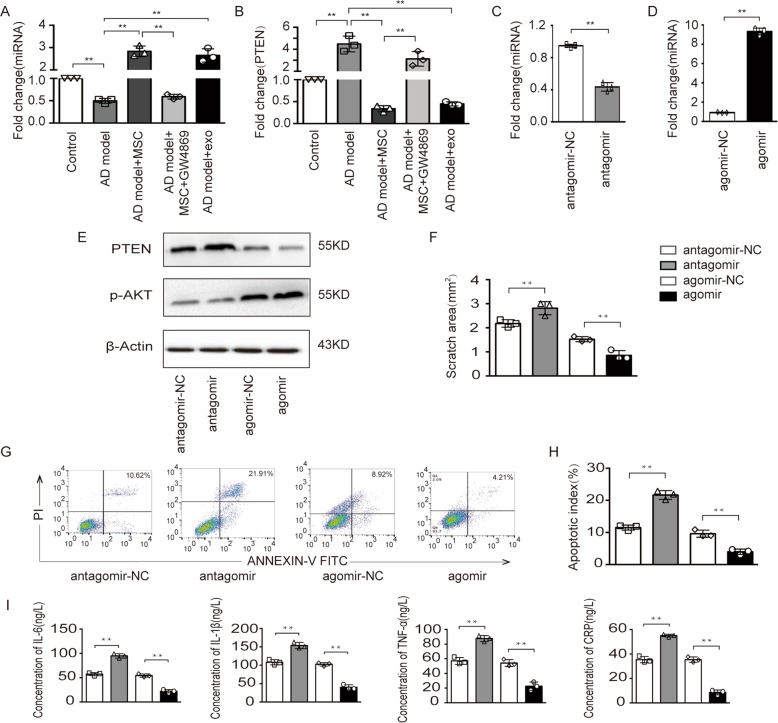
MSC-derived exosomal miR-223 reduced cell apoptosis in AD model by targeting the PTEN-PI3K/Akt pathway. **a**, **b** After coculture with MSCs, MSCs and GW4869, MSCs derived exosomes, the expression of miR-223 and PTEN levels were detected in the AD model. **c**–**h** The AD model after coculture with exosomes derived from MSCs transfected with miR-223 antagomir or agomir. The fold change of miR-223, the expression levels of PTEN and p-Akt, the scratched area, cell apoptosis and related quantitative analysis of apoptosis were detected in the AD model respectively. **i** Levels of the inflammatory cytokines IL-6, IL-1β, TNF-α, and CRP in cell supernatants from each group were measured; **p < 0.01.

The exosome encapsulating miRNA is regarded as a novel and important part of the intracellular communication mechanism, and various studies have shown that miR-223 plays a role in different cellular processes, ranging from cell cycle regulation and invasiveness to immune cell function^14^. Given its role in basic cellular functions found in our previous study, it is not surprising that miR-223 has the potential to inhibit cell apoptosis. To determine the impact of miR-223 on AD, we first detected the expression of miR-223 in the AD model, and found a time-dependent decrease in miR-223 (additional file 4A). Then, the AD model was cocultured with MSCs, and we found the increased miR-223 in the AD model. However, when MSCs in the cocultured system were pretreated with GW4869, the miR-223 level in the AD model was significantly reduced (Fig. 4a). These data indicate that the increased level of miR-223 in the AD model was largely dependent on that in MSCs. However, the levels of miR-223 in contact coculture and noncontact coculture were not significantly different. These results suggest that the effect of exosomes derived from MSCs on miR-223 expression was almost equivalent to that of MSCs.

It has been reported that one of the pathways of miR-223 participation is counteracting with PTEN^22^. Similarly, our data showed that the levels of PTEN were inversely correlated with miR-223 expression (Fig. 4b). To further explore the effects of miR-223, we inhibited miR-223 with an antagomir and overexpressed miR-223 using agomir in MSCs, and assessed the expression of miR-223 in MSCs (additional file 4B–C). The inhibition of miR-223 in MSCs resulted in a decrease in miR-223 in the AD model cocultured with exosomes (Fig. 4c), while the overexpression of miR-223 resulted in an increase in miR-223 (Fig. 4d). To further investigate this putative relationship between miR-223/PTEN and the PI3K/Akt pathway in the AD model, we examined the levels of PTEN and Akt phosphorylation. The downregulation of PTEN in the cocultured AD model with MSC-derived exosomes was enhanced when pretreated with antagomir, and reversed when pretreated with agomir, while the expression of p-AKT changed inversely (Fig. 4e). Furthermore, we evaluated the physiological effects of miR-223 on the AD model. The scratch area was decreased with the overexpression of miR-223 but increased with the downregulation by antagomir (Fig. 4f and additional file 4D). Importantly, the number of apoptotic cells was significantly smaller in the cocultured AD model with miR-223-overexpressing MSCs derived exosomes than in the other groups (Fig. 4g–h). In addition, the expression levels of the inflammatory factors IL-6, IL-1β, TNF-α, and CRP in the pretreated AD model were detected. These results showed that the expression of inflammatory factors was significantly downregulated in the miR-223-overexpressing MSC-derived exosomes-treated AD model (Fig. 4I). Model for the role of MSC-derived exosomal miR-223 in the regulation of neuronal cell apoptosis was shown in the additional file 5. Overall, these results indicated that MSC-derived exosomal miR-223 may inhibit neuronal apoptosis through the PTEN-PI3K/Akt pathway.

### Effects of exosomes derived from the serum of AD patients on neuronal apoptosis

**Fig. 5:**
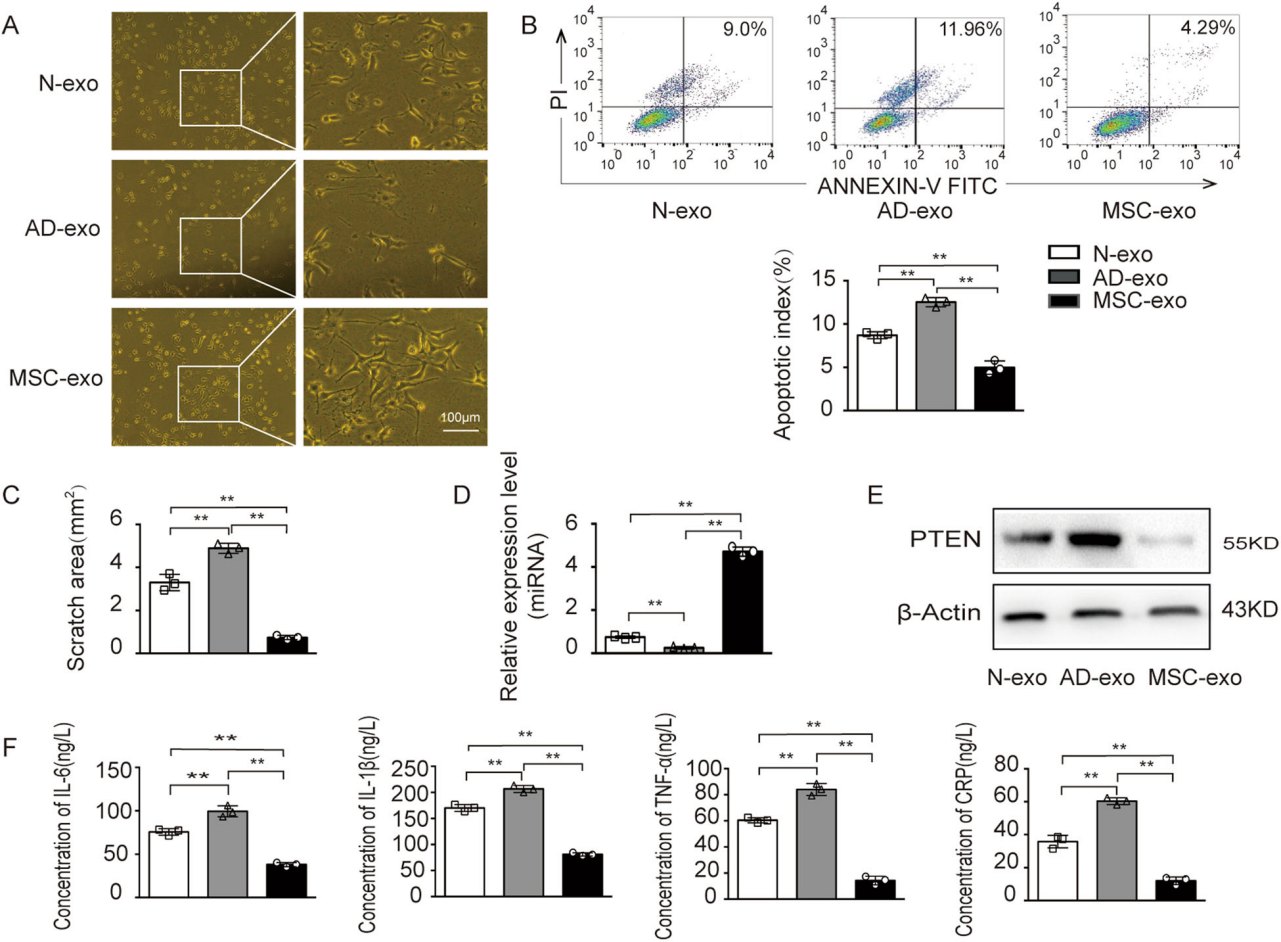
Effect of exosomes derived from the serum of AD patients on neuronal apoptosis. The AD model was treated with exosomes from healthy serum, AD serum or MSCs at a concentration of 5×10^8^ exosomes/ml for 48 h. **a**–**f** Morphological appearance, cell apoptosis, cell migration assay, miR-223 expression, PTEN expression and IL-6, IL-1β, TNF-α, and CRP levels were measured; **p < 0.01. Additional file 1: The schematic of experimental design. Additional file 2: The scratched area was quantified with ImageJ of four groups. Additional file 3: The scratched area was quantified with ImageJ of four groups. Additional file 4: **a** The level of miR-223 in the AD model of four groups. **b**, **c** The levels of miR-223 in MSCs. **d** The scratched area was quantified with ImageJ of four groups; **p < 0.01. Additional file 5: Model for the role of MSC-derived exosomal miR-223 in the regulation of neuronal cell apoptosis. Additional file 6: **a** The level of miR-223 in AD model of three groups. **b** The scratched area was quantified with ImageJ of three groups; **p < 0.01.

Since the most promising changes in exosomal cargo composition were detected in exosomes derived from human serum in our previous clinical trial, we turned to the AD model to further investigate its effects. To test whether the isolated exosomes from the serum of AD patients have any physiological effect on neuronal apoptosis, exosomes derived from human serum and MSCs were added to the AD model for contact coculture. As shown in Fig. 5a, significant changes in cell morphology were observed in the AD model cocultured with serum exosomes or MSC-derived exosomes. The AD model treated with exosomes from AD patient serum directly reduced the number of cells and shortened synapses, while that treated with exosomes derived from MSCs promoted these cell properties. Then, the physiological effects of exosomes on the cell biological function were assessed. The exosomes of AD patient serum increased apoptosis compared with those of healthy samples in the AD model, while the MSC-derived exosomes reduced apoptosis as showed (Fig. 5b). After a 48 h incubation with the AD model, all AD patient-derived exosomes significantly increased the scratch area compared to the healthy human control exosomes, while incubation of scratched cells with exosomes derived from MSCs resulted in a significantly reduced scratch area (Fig. 5c and additional file 6B). In addition, we found that the levels of exosomal miR-223 in MSCs was higher than AD patient serum (additional file 6A). And we found that the miR-223 level in the AD model pretreated with exosomes secreted by the serum of AD patients was lower than that in healthy controls and higher than that in the model pretreated with MSC-derived exosomes (Fig. 5d). The level of PETN was also verified as negatively correlated to the level of miR-223 (Fig. 5e). Moreover, the expression levels of the inflammatory factors IL-6, IL-1β, TNF-α, and CRP were significantly upregulated in the AD model pretreated with serum exosomes group but downregulated in that treated with MSC-derived exosomes group (Fig. 5F). In summary, these results indicated that exosomal cargo composition may transfer substances that correlate with the apoptosis in AD.

## Discussion

AD is a complex disease with symptoms mostly related to age-related changes, including memory loss, difficulties in planning or performing daily tasks, confusion, poor judgement and visualization, changes in personality and depression^23^. Despite all current standard treatments, the survival rate of patients is still poor, mainly because drugs are not pathologically targeted^24^. Therefore, there is a critical need for new molecular targets and approaches to treat this devastating disease.

With the cerebral hypoxic milieu being a potential feature and an irreversible factor of AD, understanding the multiple interactions between hypoxia and impaired nerve cells is of paramount significance^25^. Aβ production through the sequential proteolysis of Aβ precursor protein by BACE1 and γ-secretase complex has been universally accepted as a factor related to the pathogenesis of AD^26,27^. Evidence has shown that hypoxia exposure can augment the amyloidogenic processing of APP, resulting in the accumulation of Aβ peptides^28,29^. In addition, HIF-1α protein can directly interact with the γ-secretase complex and increase its activity in a non-transcriptional manner^30^. Our findings proved that hypoxia indeed enhanced the construction of the AD model through increased Aβ accumulation in vitro, thus promoting apoptosis and inhibiting cell migration. Our study further confirmed that hypoxia is a risk factor for AD.

Stem cell therapy has emerged as a novel and promising candidate approach for the treatment of AD, probably due to neuroprotection provided by secreting various neural factors^31^. Genetically modified and transfected MSCs have been suggested to enforce the paracrine effect of MSCs, which were successful in cerebral protection in preclinical studies^32,33^. The advantages of MSCs are more obvious compared with those of other stem cells in terms of effectiveness and convenience^34^. The composition of exosomal cargo determines the therapeutic potential of exosomes, and the fact that these vesicles were produced by cells with an already described therapeutic activity (like MSCs) increases this potential. Based on these findings, we propose that the use of MSC-derived exosomes could offer several advantages, such as a superior safety profile. In addition, the number of exosomes taken up by AD cells had a time-dependent increase, which protected AD cells from apoptosis and limited the migratory ability. Our study focused on the MSC-derived exosomes with synergistic protective effects and demonstrated the therapeutic capabilities of the MSC-derived exosomes as MSC paracrine activity effectors.

Exosomes released from activated or apoptotic cells contain specific proteins (signaling molecules, receptors, integrins, cytokines), bioactive lipids, and nucleic acids (mRNA, miRNA, small noncoding RNAs, DNA) from their progenitor cells^35,36^. MiRNAs, a class of small noncoding transcripts, are known to play important roles in development, metabolism and neural plasticity^37^. Studies have shown that dysregulation of miRNA may play a complex role in AD^38^. However, no evidence of miRNA-mediated regulation of AD progression has been demonstrated. AD is a multifactorial disease characterized by Aβ deposition, tau hyperphosphorylation, oxidative stress, cholinergic deficits, progressive synaptic loss, and neurodegeneration^39–41^. How all these typical characteristics of AD relate to each other is not clear, but AD eventually leads to nerve cell apoptosis, which is a widespread and fundamentally irreversible phenomenon. In our previous study, we identified a specific miRNA, miR-223, whose levels were downregulated in AD and remained decreased in the defined pathological areas of human AD serum during disease progression. In this study, we demonstrated that overexpression of this miRNA led to decreased apoptosis. We propose that miR-223 downregulation, observed in AD, may perturb signaling pathways associated with neuronal cell cycle, thereby causing apoptosis associated with the disease. PTEN is a validated target of the miR-223, and its downstream PI3K/Akt/mTOR signaling pathway controls neurite remodeling, cell proliferation and differentiation^42^. In our study, miR-223-mediated PTEN reduction was proven to lead to increased PI3K/Akt signal activation over time, suggesting the Akt may be involved in the miR-223/PTEN pathway. Finally, we propose that miR-223 has an important function in the pathophysiology of AD. Damage of AD cells was shown to have significantly improved following delivery of MSC-derived exosomes. In contrast, exosomes can mediate cell-to-cell propagation of toxic Aβ pathology in AD^35,43^. Our study showed that exosomes from AD patient serum exert a negative effect on cells, while exosomes from MSCs play a protective role.

Overall, our data demonstrate that the delivery of MSC-exosomes reduces the apoptosis response and promotes functional recovery in AD and revealed that MSC-derived exosomal miR-223 acted as a protective factor for AD via the PI3K/Akt signaling pathway. However, whether this pathway plays the same protective role in vivo is still unknown. Future studies on the in vivo aspect are needed for a deeper discussion. Nevertheless, our observations provide new ideas for the use of MSC exosomes as a potential treatment for patients with AD. MSC exosomes might be particularly useful for neurological conditions, in which crossing the blood–brain barrier is an important hurdle to overcome^44^. The cargo carried by exosomes, either in the CNS or periphery, has shown promise for potentially treating human neurological diseases by loading either protein or genetic content (e.g., miRNA expression)^44^. Thus, our findings have provided a rationale for the development of a ready-to-use, cell-free, and MSC-based therapeutic approach that is highly effective for the treatment of AD. This hypothesis should be validated to reinforce the clinical attractiveness of MSC-derived exosomes, making such a treatment simple, non-invasive, readily available and economically affordable.

## Supplementary information

Additional file 1

Additional file 2

Additional file 3

Additional file 4

Additional file 5

Additional file 6

Additional table
