# Correction: Mesenchymal stem cell-derived exosomal miR-223 regulates neuronal cell apoptosis

**DOI:** 10.1038/s41419-020-2583-0

**Published:** 2020-06-08

**Authors:** Hong Wei, Yuhao Xu, Qi Chen, Hui Chen, Xiaolan Zhu, Yuefeng Li

**Affiliations:** 1 Department of Central Laboratory, The Fourth Affiliated Hospital of Jiangsu University, Zhenjiang, Jiangsu 212001 China; 2 Jiangsu University, Zhenjiang, Jiangsu 212003 China; 3 Department of Neurology, The Affiliated Hospital of Jiangsu University, Zhenjiang, Jiangsu 212001 China; 4 Department of Radiology, The Affiliated Hospital of Jiangsu University, Zhenjiang, Jiangsu 212001 China

**Keywords:** Cell death in the nervous system, Mesenchymal stem cells

Correction to: *Cell Death and Disease*

10.1038/s41419-020-2490-4 published online 27 April 2020

The original version of this article did not acknowledge Yuefeng Li as a corresponding author. This has now been corrected in both the PDF and HTML versions of the article.

