# Supplementary figures and images for: Mesenchymal stem cell-derived exosomal miR-223 regulates neuronal cell apoptosis
Source: Cell Death Dis. 2020 Apr 27;11(4):290. doi: 10.1038/s41419-020-2490-4 (PMC7184756; doi:10.1038/s41419-020-2490-4)

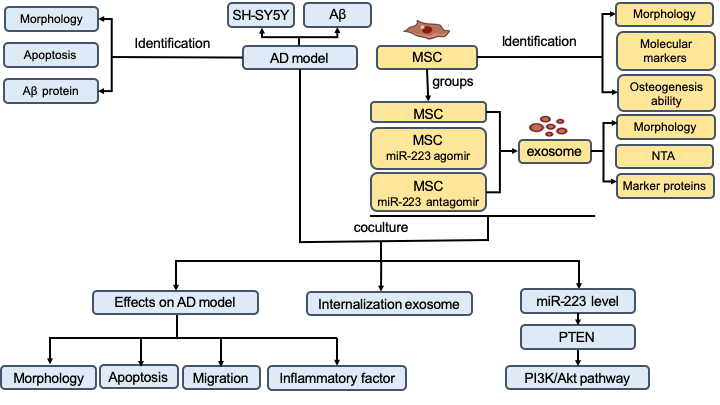

Supplement: Supplementary file 1 — Additional file 1 [file 41419_2020_2490_MOESM1_ESM.tif]

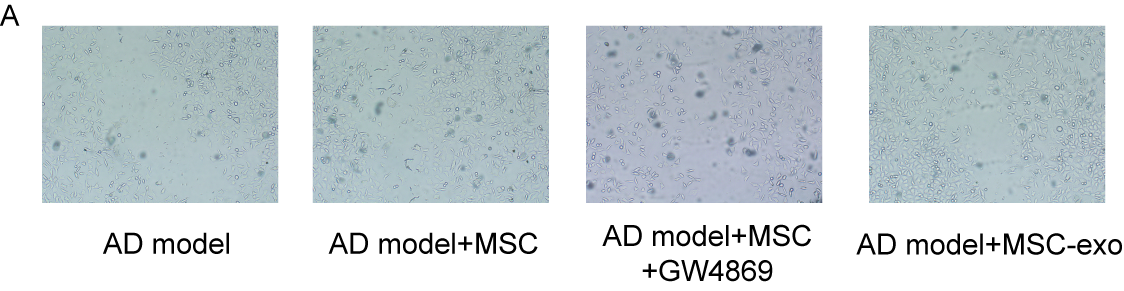

Supplement: Supplementary file 2 — Additional file 2 [file 41419_2020_2490_MOESM2_ESM.tif]

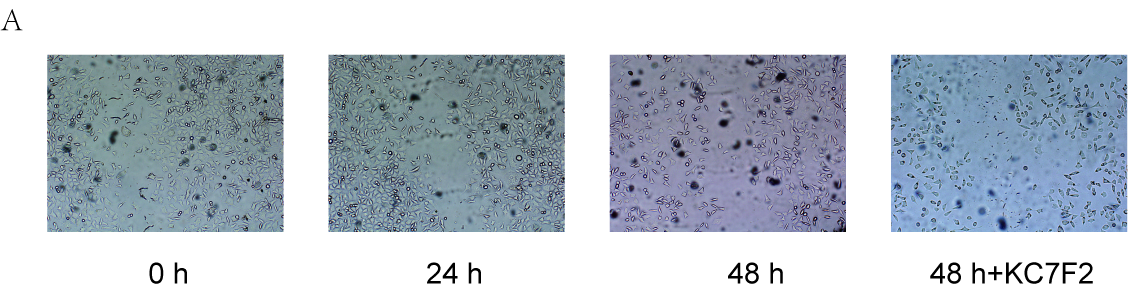

Supplement: Supplementary file 3 — Additional file 3 [file 41419_2020_2490_MOESM3_ESM.tif]

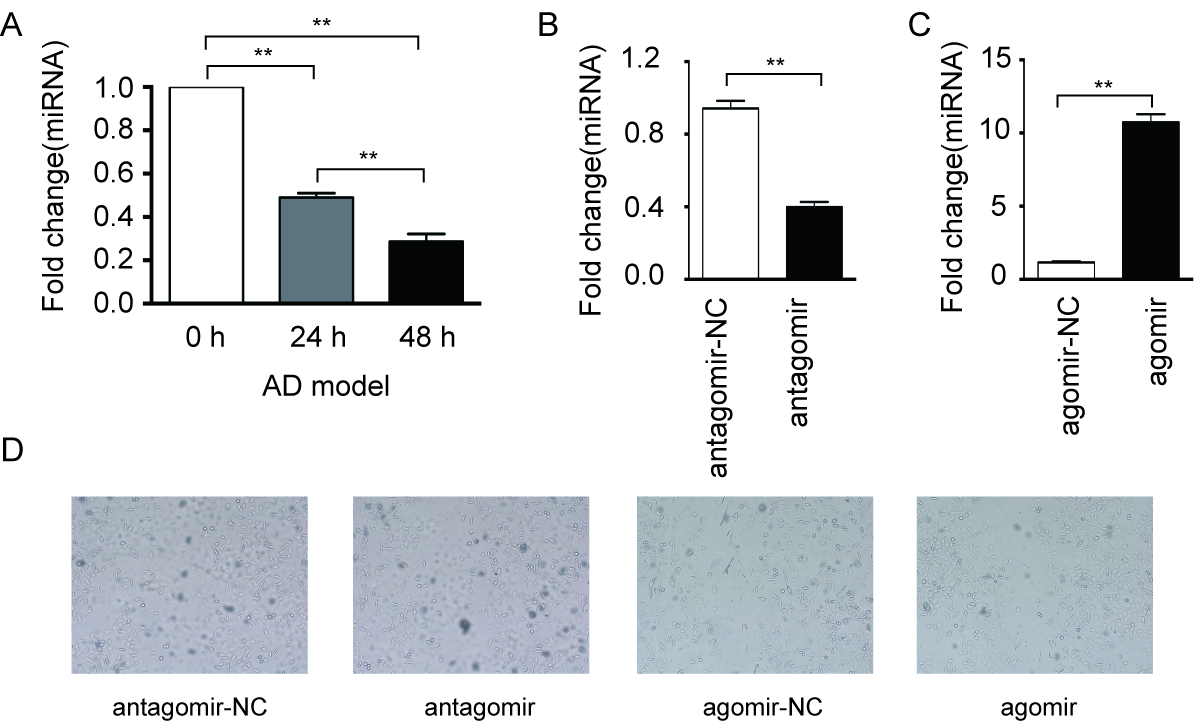

Supplement: Supplementary file 4 — Additional file 4 [file 41419_2020_2490_MOESM4_ESM.tif]

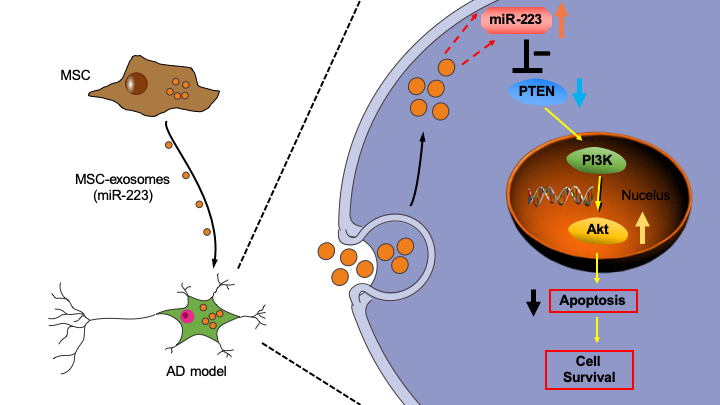

Supplement: Supplementary file 5 — Additional file 5 [file 41419_2020_2490_MOESM5_ESM.tif]

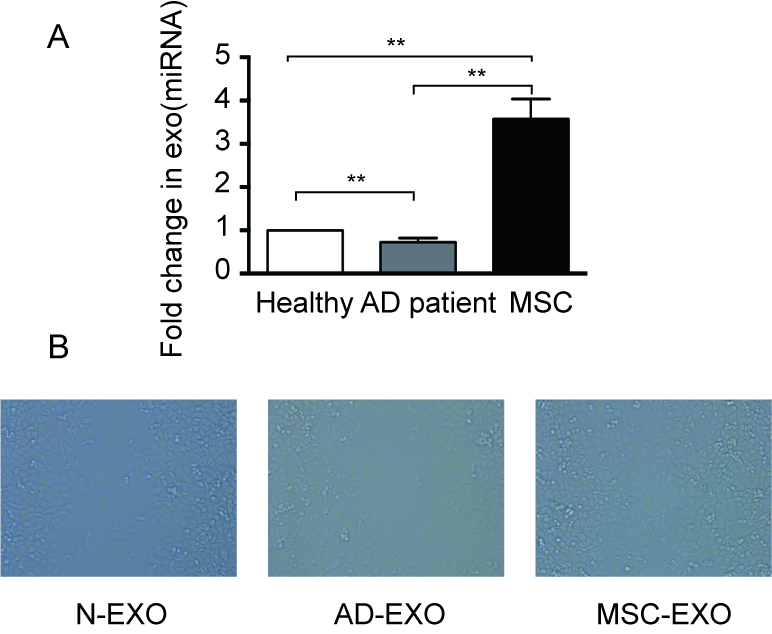

Supplement: Supplementary file 6 — Additional file 6 [file 41419_2020_2490_MOESM6_ESM.tif]
